# Pharmacogenomic and Pharmacomicrobiomic Aspects of Drugs of Abuse

**DOI:** 10.3390/genes16040403

**Published:** 2025-03-30

**Authors:** Alejandro Borrego-Ruiz, Juan J. Borrego

**Affiliations:** 1 Departamento de Psicología Social y de las Organizaciones, Universidad Nacional de Educación a Distancia (UNED), 28040 Madrid, Spain; 2 Departamento de Microbiología, Universidad de Málaga, 29071 Málaga, Spain; jjborrego@uma.es

**Keywords:** pharmacogenomics, pharmacomicrobiomics, drugs of abuse, substance use disorders, gut microbiome

## Abstract

Background/Objectives: This review examines the role of pharmacogenomics in individual responses to the pharmacotherapy of various drugs of abuse, including alcohol, cocaine, and opioids, to identify genetic variants that contribute to variability in substance use disorder treatment outcomes. In addition, it explores the pharmacomicrobiomic aspects of substance use, highlighting the impact of the gut microbiome on bioavailability, drug metabolism, pharmacodynamics, and pharmacokinetics. Results: Research on pharmacogenetics has identified several promising genetic variants that may contribute to the individual variability in responses to existing pharmacotherapies for substance addiction. However, the interpretation of these findings remains limited. It is estimated that genetic factors may account for 20–95% of the variability in individual drug responses. Therefore, genetic factors alone cannot fully explain the differences in drug responses, and factors such as gut microbiome diversity may also play a significant role. Drug microbial biotransformation is produced by microbial exoenzymes that convert low molecular weight organic compounds into analogous compounds by oxidation, reduction, hydrolysis, condensation, isomerization, unsaturation, or by the introduction of heteroatoms. Despite significant advances in pharmacomicrobiomics, challenges persist including the lack of standardized methodologies, inter-individual variability, limited understanding of drug biotransformation mechanisms, and the need for large-scale validation studies to develop microbiota-based biomarkers for clinical use. Conclusions: Progress in the pharmacogenomics of substance use disorders has provided biological insights into the pharmacological needs associated with common genetic variants in drug-metabolizing enzymes. The gut microbiome and its metabolites play a pivotal role in various stages of drug addiction including seeking, reward, and biotransformation. Therefore, integrating pharmacogenomics with pharmacomicrobiomics will form a crucial foundation for significant advances in precision and personalized medicine.

## 1. Introduction

Initial substance use is often driven by curiosity, but with reinforcement, such behavior can evolve into a habitual pattern, ultimately leading to addiction [1]. This progression is of particular public health concern, as evidenced by the recent fentanyl crisis [2]. Substance addiction is characterized by specific psychological symptoms, such as intense cravings or repeated failed attempts to regulate consumption, and by physiological manifestations including tolerance and withdrawal syndrome [3]. The regular consumption of addictive substances can exert a profound impact on both physical well-being and socio-emotional stability, heightening the risk for polysubstance dependence, medical emergencies, and dysfunction in family dynamics [4]. Furthermore, substance use has been linked to various psychiatric conditions and their associated symptoms including major depressive disorder, suicidal thoughts and behaviors, schizophrenia, bipolar disorder, borderline personality disorder, anxiety disorders, and antisocial personality disorder [5]. Thus, the potential individual, familial, and societal consequences of substance abuse underscore the critical need to deepen our understanding of the pharmacogenetic mechanisms involved in addiction. Moreover, developing novel strategies to enhance the metabolism and clearance of these substances is essential for mitigating their harmful effects [6].

Pharmacogenetics constitutes the study of how genetic variability influences drug responses and adverse reactions, aiming to optimize individualized treatment approaches. Understanding how genetic differences impact drug responses can also shed light on fundamental pharmacological and pathological mechanisms [7]. Early pharmacogenetic research primarily targeted candidate genes, chosen based on prior knowledge of the pharmacokinetic and pharmacodynamic properties of psychotropic medications [8]. Genetic diversity in humans is largely attributed to single nucleotide polymorphisms (SNPs), which involve single-base alterations in the DNA sequence including insertions, deletions, and copy number variations [9].

The development of chip-based microarray technology, capable of analyzing thousands of genetic polymorphisms across the genome, has significantly advanced genetic association studies and facilitated the emergence of genome-wide association studies (GWASs). This technological progress has led to the evolution of pharmacogenomics, a field that extends beyond single-gene analyses to encompass the entire genome, enabling the accurate genotyping of between 500,000 and over 4 million SNPs in a cost-efficient manner. As a rapidly expanding discipline, pharmacogenomics aims to identify genetic markers that can guide clinicians in selecting safe and effective treatments customized to individual patients [10]. Consequently, pharmacogenomic testing holds great promise for optimizing drug therapy across a broad spectrum of medical conditions [11].

The combined genetic material of a host organism and its associated microbiota has led to the introduction of the hologenome concept [12]. According to the holobiont theory of evolution, natural selection can act not only on the host genome, but also on the metagenome of the microbiota, shaping them into a cohesive functional unit [13]. Stilling et al. [14] introduced the term holoepigenome to describe the epigenetic characteristics and heritability of the gut microbiome (GM). More recently, Pepke et al. [15] expanded this definition, describing the holoepigenome as the complete set of epigenetic modifications and their complex interactions within the host genome.

The GM constitutes a highly diverse and complex biological ecosystem, which is estimated to encompass over 5 million distinct genes and approximately 100 trillion cells [16,17]. Established at birth, the GM undergoes continuous modifications throughout the lifespan of an individual, dynamically reflecting the health status at different life stages [18,19]. These fluctuations can be transient or permanent and are influenced by factors such as diet, stress, hormonal changes, and environmental exposures [20,21]. Beyond its crucial role in metabolic processes, the GM also contributes to immune regulation and behavioral traits, while significantly impacting drug metabolism through biotransformation mechanisms including hydrolysis, demethylation, deamination, and various reactive transformations [22]. Since the 20th century, research has explored the influence of the GM on drug metabolism and efficacy, revealing its capacity to activate, inactivate, or modify the toxicity of drugs and xenobiotics [23].

Pharmacomicrobiomics examines how variations in the microbiome influence the disposition, efficacy, and response to drugs and xenobiotics [24]. More specifically, this field explores the ways in which intra- and inter-individual differences in microbial composition affect drug action, pharmacokinetics, pharmacodynamics, metabolism, bioavailability, therapeutic outcomes, and potential toxicity, rather than drug–drug interactions with specific microorganisms [22,25,26]. Research has identified several mechanisms underlying drug–microbiota interactions including biotransformation (where microbes metabolize drugs), bioaccumulation (where drugs are stored within microbial cells), and the direct modulation of microbial growth by pharmacological compounds [27]. In this regard, several chemical compounds have been reported to be degraded by bacterial species. For example, the degradation of digoxin, a cardiac glycoside, by *Eggerthella lenta* and *Lactiplantibacillus pentosus* [28,29], the degradation of salicin, a dietary plant β-glucoside, by *Lactobacillus acidophilus* [30], or prontosil by *Clostridium* and *Eubacterium* species [23], which uses several bacterial enzymes such as β-glucuronidase [31], phospho-β-glucosidase [32], and nitroreductase [33]. Table 1 illustrates the key differences between pharmacogenomics and pharmacomicrobiomics (according to [34,35]).

In this narrative review, we examined the role of pharmacogenomics in modulating individual responses to the pharmacotherapy of various drugs of abuse including alcohol, cocaine, and opioids. By identifying promising genetic variants, we have highlighted potential contributors to individual variability in treatment outcomes for substance use disorder (SUD). However, additional factors such as GM diversity may also play an important role in pharmacotherapy efficacy. For this reason, we explored the pharmacomicrobiomic aspects related to substance use as a means to understand the complex interactions between the GM, the host, and drugs, which possess important implications for bioavailability, drug metabolism, pharmacodynamics, pharmacokinetics, and the risk of drug-induced toxicity.

## 2. The Role of Pharmacogenomics in Drug Response

**Figure 1:**
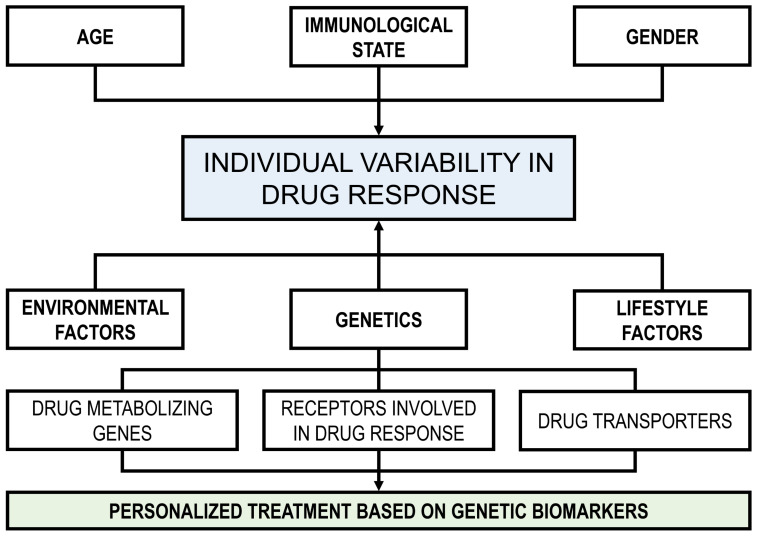
Factors affecting individual variability in drug response and personalized treatment based on pharmacogenomics.

Significant inter-individual differences in the efficacy and safety of numerous therapeutic agents arise from genetically regulated polymorphisms affecting drug-metabolizing enzymes, transport proteins, and receptor targets. The emergence of pharmacogenomics as a distinct field has promoted advancements in pharmacogenetics by uncovering the genetic foundations of these inherited differences at the genomic level, providing deeper insights into the mechanisms underlying drug response variability [10,36]. Pharmacogenomics has given rise to the practice of “precision medicine”, which combines pharmacology (the study of drugs) and genomics (the study of genes and their functions) to offer a more customized approach [37]. In the context of drug addiction, pharmacogenetics studies the genetic variation that affects treatment response, defined as treatment outcomes or adverse effects [38]. However, pharmacogenetics has only recently been applied to the study of addiction treatment for alcoholism and opioid addiction. In contrast, there are currently no approved therapies for psychostimulants (e.g., cocaine and methamphetamine addiction). Figure 1 presents several factors affecting the individual variability in drug response and personalized treatment based on pharmacogenomics.

Genetic variation can affect gene expression at multiple levels including transcriptional regulation, mRNA splicing and stability, and protein translation, stability, and function. These variations can subsequently impact protein folding, structure, and biological processes such as enzymatic activity, binding affinity, and stability [9,38]. Polymorphisms in genes that encode key components of pathways related to SUDs may contribute to individual differences in response to addiction treatments. These variations are typically polygenic, involving multiple genes, but could also be oligogenic, involving a small number of genes that have a dominant influence on the response [10,38].

Research has shown that genetic factors are critical in determining individual variations in drug therapy outcomes and significantly contribute to adverse drug reactions [39,40,41]. SNPs refer to variations in DNA sequences caused by the alteration of a single nucleotide at the genomic level [42]. These genetic variations can influence drug metabolism and responses, making SNPs a key focus in pharmacogenomic research. For example, Salvador-Martín et al. [43] identified that HLA DNA variants (rs2395185 and rs2097432) were linked to long-term responses to anti-tumor necrosis factor therapy in Spanish children with inflammatory bowel disease, highlighting the important role of SNPs in drug response.

The Pharmacogenomics Knowledge Base (PharmGKB: http://www.pharmgkb.org accessed on 17 February 2025), established in 2000, serves as a comprehensive resource for pharmacogenomics data [44]. As of September 2023, PharmGKB had annotated 766 drugs, 201 clinical guidelines, 933 drug labels, and 428 FDA drug labels. It provides detailed information on the relationships between drugs, genes, and diseases, offering researchers valuable insights into genetic variation annotations, drug-centric pathways, key pharmacogenes, and various diseases [45]. Table 2 shows the genetics of several approved and investigational pharmacotherapies for addictions and the genetic variants.

Drug addiction is a prevalent, polygenic, chronic, and relapsing brain disorder that could benefit from pharmacotherapies more precisely defined through molecular-genetic approaches. The pharmacogenetic studies reviewed identified several promising genetic variants that may contribute to the individual variability in responses to existing pharmacotherapies for drug addiction. However, the interpretation of these findings remains limited. It is estimated that genetic factors may account for 20–95% of the variability in individual drug responses [35]. Therefore, genetic factors alone cannot fully explain the differences in drug responses, and other factors such as GM diversity may also be important [74].

## 3. Influence of Drugs of Abuse on the Gut Microbiome

An expanding body of research has revealed that various drugs of abuse affect the GM, both in animal models and in humans. The bidirectional drug–microbe interaction may be pivotal for future drug development and clinical application. In this section, we review the major studies regarding the influence of drugs of abuse, such as alcohol, psychostimulants, opioids, cannabinoids, and nicotine, on the composition of the GM.

### 3.1. Alcohol

Alcohol is a depressant and is one of the oldest and most widely used recreational substances. Its primary mechanism of action is to increase the activity of γ-aminobutyric acid (GABA), which is a major inhibitory neurotransmitter in the brain, and this increased activity results in central nervous system depression [75]. Another primary target of alcohol in the neural system is the N-methyl-D-aspartate (NMDA) receptor, a key component of the glutamate receptor family that plays a central role in several brain functions including learning, memory, and synaptic plasticity [76].

In a mouse model of alcoholic liver disease, members of the phyla Bacteroidota and Verrucomicrobiota were increased in alcohol-fed mice compared with a relative predominance of members of the phylum Bacillota in the control mice [77]. Exposure of mice to 4 weeks of chronic intermittent vaporized ethanol significantly altered the GM, increasing the levels of *Alistipes* and decreasing *Clostridium* IV, *Dorea*, and *Coprococcus* [78].

Several human studies have shown that alcohol consumption leads to an increase in the abundance of the members of the phylum Pseudomonadota and to a decrease in the members of the phylum Bacteroidota [79,80]. At the genus level, increases in the abundance of *Bacteroides*, *Clostridium*, *Holdemania*, *Sutterella*, and *Streptococcus* have been reported as well as decreases in the genera *Akkermansia* and *Faecalibacterium* [79,80,81]. Recently, Du et al. [82] found that the microbial diversity and composition of the GM were dysregulated in patients with alcohol use disorder (AUD), with a decrease in α-diversity and an increase in β-diversity indices including a decreased abundance in the members of the family *Lachnospiracea* and in the genera *Bacteroides*, *Dialister*, *Faecalibacterium*, and *Gemmiger*, and increased levels in the genera *Coprobacillus*, *Clostridium*, *Escherichia*, *Fusobacterium*, *Gemella*, *Megamonas*, *Prevotella*, and *Rothia*. In contrast, patients with alcoholic cirrhosis showed an increase in the abundance of *Escherichia/Shigella* and *Prevotella*, and a decrease in the genera *Blautia* and *Faecalibacterium* [83].

### 3.2. Psychostimulants

Psychostimulants are indirect sympathomimetic substances with a high potential for misuse and addiction, which increase the release of dopamine and enhance synaptic transmission as well as the availability of monoamine neurotransmitters at the synapse such as dopamine, norepinephrine, and serotonin [84]. These pharmacological compounds are recognized for their invigorating properties and extensive global utilization including both in medical and non-medical contexts for performance enhancement and recreational purposes [85]. Commonly abused psychostimulants include amphetamine, methamphetamine, MDMA (ecstasy), methylphenidate, and cocaine [86].

The results regarding GM composition in relation to psychostimulant use are diverse due to the varying chemical properties and receptor interactions of these substances. In preclinical studies, the fecal microbial diversity is slightly higher in methamphetamine-treated rats, with a decrease in the propionate-producing genus *Phascolarctobacterium* and an increase in members of the family *Ruminococcaceae* [87]. In humans, the methamphetamine use was associated with increases in the genera *Finegoldia*, *Fusobacterium*, *Parvimonas*, *Peptoniphilus*, *Peptostreptococcus*, *Porphyromonas*, *Streptobacillus*, and *Streptococcus*, and with decreases in *Bacteroides*, *Butyricicoccus*, *Faecalibacterium*, and *Succinivibrio* [88]. Furthermore, in the GM of methamphetamine users, Yang et al. [89] reported significant decreases in the abundance of members of the phylum Myxococcota (formerly class Deltaproteobacteria) and the family *Bacteroidaceae* as well as increases in the abundance of Sphingomonadales, Xanthomonadales, *Romboutsia*, and *Lachnospiraceae*. In a recent study, He et al. [90] showed significant changes in the GM composition of methamphetamine use disorder (MUD) and methamphetamine causal use (MCU) individuals compared with the controls, with a dominance of *Bacteroides*, *Blautia*, *Coprococcus*, *Dialister*, *Faecalibacterium*, *Lachnospira*, *Megamonas*, *Prevotella*, *Roseburia*, and *Ruminococcus*. These authors also reported a higher abundance of the genera *Clostridium*, *Devosia*, *Dorea*, and *Halomonas* in the GM of the MUD group compared with the MCU group.

In the case of cocaine, Volpe et al. [91] found that the use of this substance was associated with an increased and decreased abundance of members of the phyla Bacteroidota and Bacillota, respectively. Gerace et al. [92] reported that the dominant genera in stool samples from cocaine users were *Bacteroides*, *Bifidobacterium*, *Blautia*, *Collinsella*, and *Faecalibacterium*. Comparing the GM of cocaine users to non-users, these authors revealed a significant reduction in the α-diversity of cocaine users. Moreover, they also identified higher fecal abundances of *Erysipelotrichaceae*, *[Clostridium]_innocuum_group* spp., *[Ruminococcus]_torques_group* spp., *Blautia* spp., *Clostridium_sensu_stricto* spp., *Collinsella* spp., *Dorea.* spp., *Escherichia-Shigella* spp., *Eubacterium* spp., *Holdemanella* spp., *Megamonas* spp., *Peptococcus* spp., *Romboutsia* spp., *Rothia* spp., *Senegalimassilia* spp., *Streptococcus* spp., and *Turicibacter* spp., in the drug-use group. In contrast, these authors found reduced levels of *Christensenellaceae*, *Desulfovibrionaceae*, *Lachnospiraceae*, *[Eubacterium]_ventriosum_group* spp., *[Eubacterium]_xylanophilum_group* spp., *Alistipes.* spp., *Bacteroides.* spp., *Barnesiella* spp., *Coprobacter* spp., *GCA-900066575* spp., *Odoribacter* spp., *Oscillospira* spp., *Paraprevotella* spp., *Parasutterella* spp., *Prevotellaceae_NK3B31_group* spp., *Sutterella* spp., and *UCG-002* spp. [92].

### 3.3. Opioids

Opioids are a group of powerful, medically important substances that are chemically related to the natural compounds found in opium, which is derived from the opium poppy plant (*Papaver somniferum*). These substances are recognized for their dual effects regarding strong pain relief and sedation [93]. However, they also carry a significant risk of addiction and abuse [94]. Opioids work by binding to specific receptors on the surface of various cells, known as opiate receptors (μ, κ, and δ).

Opioids used as analgesics diminish the intensity and unpleasant perception of pain through the activation of specific G protein-coupled receptors in the brain, spinal cord, and peripheral nervous system [95]. Acting as agonists at the opioid receptors, these compounds reduce neuronal excitability and inhibit the release of pain neurotransmitters [96]. In vitro studies have shown that opioids present conflicting results regarding their antimicrobial activity. On the other hand, morphine does not appear to have an inhibitory effect on microbial strains [97]. Other opioids, such as bupivacaine, pethidine, tramadol, fentanyl, and methadone, have shown potent bactericidal activity in vitro against *Bacillus cereus*, *Enterococcus faecalis*, *Escherichia coli*, *Klebsiella pneumoniae*, *Pseudomonas aeruginosa*, *Serratia marcescens*, *Staphylococcus aureus*, *S. epidermidis*, *Streptococcus pneumoniae*, and *S. pyogenes* [97,98,99,100,101].

One of the many debilitating side effects of chronic opioid use is opioid-induced intestinal dysfunction and bacterial translocation, both in the bacterial metabolite profile and in the intestinal barrier integrity [102,103]. In preclinical studies, morphine induced changes in the GM composition of a mouse model associated with a significant increase in pathogenic bacteria such as *Clostridium*, *Enterococcus*, *Flavobacterium*, *Fusobacterium*, and *Sutterella* [104]. In another study in mice, subcutaneous injections of tramadol inhibited the growth of *S. aureus* but did not eliminate *P. aeruginosa* [105]. However, the relationship between GM dysbiosis and opioid use in humans has not been well-studied [106]. In a cohort of cirrhotic patients, multiple chronic opioid use (e.g., hydromorphone, fentanyl, methadone, morphine, oxycodone, percocet, tramadol, and combinations of these drugs) was associated with significant changes in GM composition, with a lower relative abundance of members of the families *Bacteroidaceae* and *Ruminococcaceae*, and of the genus *Bacteroides* [107].

### 3.4. Cannabinoids

Cannabinoids are compounds derived from the cannabis plant (*Cannabis sativa* and *C. indica*), which is widely used worldwide for both cultural and medical purposes. These substances have been the subject of extensive research due to their low potential to induce dependence, their relatively mild adverse health effects, and numerous potential therapeutic benefits [108,109]. In vitro studies have shown that cannabis exerts an antimicrobial activity against a broad range of microorganisms including *Bacillus subtilis*, *S. aureus*, *E. coli*, and *P. aeruginosa* [110]; methicillin-resistant *S. aureus* [111]; and *Clostridium* spp., *Enterococcus* spp., *Pseudomonas* spp., and *Pectobacterium* spp. [112].

Given that the main method of cannabis use in humans is via smoking, few studies have been conducted on the effect of this substance on the GM. Mehrpouya-Bahrami et al. [113] found that CB1 antagonism reduced intestinal permeability and altered the GM including an increase in the abundance of *Akkermansia muciniphila* and a decrease in the members of the families *Lachnospiraceae* and *Erysipelotrichaceae*. In a cohort study comparing cannabis users and non-users, Panee et al. [114] reported that the abundance of *Prevotella* and *Bacteroides* was inversely correlated among participants. In this regard, the ratio of *Prevotella/Bacteroides* was 13 times higher in the non-users. Vijay et al. [115] reported a positive association of endocannabinoids with bacterial α-diversity as well as with short-chain fatty acid (SCFA)-producers such as *Bifidobacterium*, *Coprococcus*, and *Faecalibacterium* while noting a negative association with *Collinsella* and *Escherichia-Shigella*.

### 3.5. Nicotine

Nicotine, which is one of the primary components of tobacco, has all the features of an addictive substance [116]. It modulates dopamine activity in the midbrain, particularly in the mesolimbic system, which promotes the development and maintenance of rewarding behaviors [117]. Two in vitro studies have investigated the antimicrobial activity of nicotine. The psychotropic compound was active against *Listeria monocytogenes* and viridans streptococci (*S. viridans*) [118] as well as against *E. faecalis*, *E. coli*, and *P. aeruginosa* [119].

Several clinical studies have been conducted on the effect of tobacco on the GM. For instance, smoking cessation determines profound changes in the GM, with an increase in microbial diversity and the abundance of the Bacillota and Actinomycetota phyla and a decrease in members of the Bacteroidota and Pseudomonadota phyla [120]. In addition, Vogtmann et al. [121], investigating the effect of smoking on the upper gastrointestinal microbiome, reported that smoking was associated with an increase in both α- and β-diversity, and the species *Dialister invisus* and *Megasphaera micronuciformis* were the most frequently detected in smokers. Moreover, a decrease in microbial population diversity, with a reduction in the genera *Collinsella*, *Enterorhabdus*, *Faecalibacterium*, and *Gordonibacter*, was found in Crohn’s disease patients who smoked [122].

In a study including never, former, and current smokers, Lee et al. [123] found no difference in α-diversity among the three groups. However, bacterial β-diversity exhibited significant differences. In current smokers, it increased the abundance of the phylum Bacteroidota while it decreased Bacillota and Pseudomonadota compared with those who never smoked. Conversely, there were no differences between the former and those who had never smoked. In a review study, Savin et al. [124] reported increases in members of the phyla Pseudomonadota and Bacteroidota, and in the genera *Bacteroides*, *Clostridium*, and *Prevotella*. They also reported a decrease in the abundance of Actinomycetota and Bacillota phyla, and in the genera *Bifidobacterium* and *Lactococcus*.

Shanahan et al. [125] reported that smokers had lower bacterial diversity in the upper small intestinal mucosa compared with non-smokers. The GM in smokers showed a higher relative abundance of members of the phylum Bacillota (genera *Streptococcus* and *Veillonella*) and Actinomycetota (genus *Rothia*) as well as lower levels of Bacteroidota (genus *Prevotella*) and Pseudomonadota (genus *Neisseria*). These results are in contrast to those of Stewart et al. [126], who reported an increase in the abundance of the genera *Clostridium* and *Prevotella* and a decrease in the genus *Bacteroides*. In addition, Nolan-Kenney et al. [127] found a relatively higher abundance of the *Catenibacterium* genus and of members belonging to the family *Erysipelotrichaceae* in current smokers. On the other hand, Lin et al. [128] reported that the relative abundance of the phyla Bacteroidota and Bacillota, and of more than 40 genera, were altered with cigarette and alcohol consumption, with the most significant decrease in the abundance of members of the family *Ruminococcaceae*. More recently, Antinozzi et al. [129] reviewed the influence of traditional and electronic cigarette smoking on the human gut microbiota. The genus *Prevotella* appeared to be significantly increased in current and former cigarette smokers, but not in electronic cigarette users. Furthermore, the genus *Desulfovibrio* (phylum *Thermodesulfobacteriota*) showed a progressive increase correlated with cigarette consumption (measured in packs per year). Moreover, Alphaproteobacteria levels were higher in the current smokers compared with those who had never smoked.

## 4. Gut Microbiome and Substance Abuse

The initiation of drug use is strongly linked to environmental and social factors [130], with curiosity and expansion motives being the primary intrinsic drivers [1]. The existence of a positive association between the frequency of addictive substance use and the level of sensation seeking has been established [131]. In this regard, the GM may play a role in the initial stage of SUDs, particularly in the search and preference for novel substances.

Individuals with high novelty-seeking tendencies are particularly sensitive to novel, packaged addictive drugs. Those with cocaine and methamphetamine use disorders exhibit significantly greater drug-seeking behavior compared with healthy controls, which may contribute to sustained drug use [132]. Moreover, novelty-seeking traits have been positively correlated with relapse rates among cocaine users [133,134]. Interestingly, alterations in the GM can significantly enhance novelty-seeking behavior in animal models. Studies have shown that modifying the GM, either by administering probiotics or suppressing bacterial populations with antibiotics, leads to a marked increase in cocaine preference. This suggests that the GM and its metabolites may influence an individual’s predisposition to substances such as cocaine and morphine [135,136,137,138]. In addition, the GM may indirectly increase the likelihood of addictive drug use through its interaction with ghrelin, a hormone produced in the gastrointestinal tract. Positive correlations have been observed between ghrelin levels and the abundance of *Clostridium* and *Ruminococcus*. On the other hand, an increased Bacteroidota/Bacillota ratio as well as higher levels of *Faecalibacterium* and *Prevotellaceae* have been negatively associated with ghrelin concentrations [139]. Rahat-Rozenbloom et al. [140] reported that ghrelin secretion decreases as SCFA production in the GM increases, and this upregulation of the ghrelin system may further contribute to drug cravings [141]. In summary, the GM and its metabolites may influence the ghrelin secretion levels, potentially affecting external behaviors such as novelty seeking, which in turn could impact an individual’s susceptibility to addictive substances.

The GM and its metabolites play a direct role in the reward mechanisms of addictive substances. The rewarding effect is triggered by the release of dopamine and other neurotransmitters that induce euphoria [142]. Through the GM–brain axis, the GM can influence brain regions involved in dopaminergic neurotransmission such as the ventral tegmental area and nucleus accumbens [143]. Lee et al. [138] demonstrated a causal relationship between GM alterations and impaired reward responses in antibiotic-treated mice. Notably, normal reward behavior was restored through fecal microbial transplantation, highlighting the role of the GM in modulating reward sensitivity. Similarly, GM and its metabolites have been shown to be essential for morphine-induced reward mechanisms [136]. In addition to direct effects on dopaminergic pathways, the GM and its metabolites may indirectly regulate drug-related reward circuits via glucagon-like peptide 1 (GLP-1). GLP-1 and its analogs modulate abnormal reward effects induced by substances such as cocaine and amphetamines, with GLP-1 receptors (GLP-1R) being highly expressed in reward-related brain regions [144]. Research indicates that GLP-1 and GLP-1R enhance behavioral responses to cocaine in mice, while the loss of GLP-1R influences anxiety-related behaviors [145]. Changes in GLP-1 levels have been positively correlated with the abundance of Actinomycetota and Bacillota members, whereas Bacteroidota and *Blautia* spp. show an inverse correlation with the GLP-1 levels [146]. Furthermore, GM-derived SCFAs stimulate GLP-1 release and elevate its plasma concentration via the phospholipase C signaling pathway [147]. Overall, during relapse, the GM contributes to the enhancement of drug reward effects both directly, through dopaminergic modulation, and indirectly, via GLP-1 regulation.

Drug-induced epigenetic modifications have been identified as key mediators of genetic changes in brain regions involved in reward processing and drug-seeking behavior. These modifications also contribute to the development of tolerance to drug use [148]. The GM and its metabolites are known to influence the epigenetic landscape under various physiological conditions [149,150]. Intestinal microorganisms produce a diverse array of bioactive small molecules including SCFAs and other microbial by-products such as β-hydroxybutyrate, folate, and B vitamins. These compounds play crucial roles in both physiological regulation and epigenetic modifications [151].

SCFAs are critical inhibitors of histone deacetylases (HDACs), enzymes that remove acetyl groups from histones, thereby leading to chromatin condensation and gene silencing [152,153]. Furthermore, SCFAs influence histone decrotonylation and acylation [154] and act as donor substrates for histone acetyltransferases (HATs) [155]. Beyond histone modifications, SCFAs also modulate the activity of ten-eleven translocation (TET) methylcytosine dioxygenases, key enzymes involved in DNA demethylation. When targeted to gene promoters, TET enzymes generally enhance gene transcription, further highlighting the epigenetic regulatory role of SCFAs [156].

Other microbial metabolites, such as B vitamins and methionine, serve as donors of methyl and acetyl groups by producing S-adenosylmethionine (SAM), which acts as a substrate for DNA methyltransferases (DNMTs) and histone methyltransferases (HMTs) [157]. While folate (vitamin B9) is primarily obtained through the diet, it can also be synthesized by various bacterial species including *Bifidobacterium bifidum*, *B. longum*, and *Limosilactobacillus reuteri* [158]. Most B vitamins, including biotin (vitamin B7), cobalamin (vitamin B12), niacin (vitamin B3), pantothenic acid (vitamin B5), and riboflavin (vitamin B2), are either produced by the microbiota or acquired through the diet. These vitamins are essential cofactors in the folate cycle, which is involved in key epigenetic processes such as DNA methylation, histone deacetylation, and histone acetylation [159,160,161,162]. All of these epigenetic modifications play a role in various processes related to the response to drugs of abuse [148].

## 5. The Role of the Gut Microbiome in Drug Pharmacokinetics

Pharmacokinetics is a subfield of pharmacology focused on determining the fate of xenobiotics once introduced into a living organism. Drug pharmacokinetics encompass four key processes: absorption, distribution, metabolism, and excretion (ADME) [163]. Absorption refers to the transfer of a substance from its administration site into the bloodstream [164]. Currently, the influence of the GM on drug absorption remains largely unexplored, and the limited available studies did not focus on drugs of abuse [165].

The GM can also impact the bioavailability of drugs without altering the drug chemical structure by modifying its absorption, solubility, and transport [166]. One key mechanism involves the production of secondary bile acids that can hydrolyze conjugated bile acids to their free form [167]. In particular, the GM can modulate the drug pharmacokinetics by regulating P-glycoprotein (MDR1 or ABCB1), a pivotal barrier to cellular drug uptake [168]. In addition, the GM can also affect OATP2B1, a transporter that mediates the absorption of various drugs [169]. Furthermore, SCFAs produced by the GM [170] have a potential role in restoring intestinal permeability and influencing the passive absorption of drugs in intestinal epithelial cells [170,171].

Drugs undergo metabolism through various processes, including conjugation, reduction, oxidation, isomerization, hydrolysis, and condensation, with the liver serving as the primary site of drug metabolism or biotransformation in humans [172]. According to Lakshmanan [173], most drugs are metabolized in two phases. In the first, the non-synthetic reactions introduce a hydrophilic group into the drug molecule, primarily through oxidation, reduction, and hydrolysis. Oxidation is catalyzed by a group of liver enzymes known collectively as cytochrome P450 (Cyp-450). Reduction occurs in drug molecules containing reducible nitro, azo, alkene, aldehyde, or ketone groups, and is catalyzed by specific enzymes called reductases. In turn, hydrolysis takes place in drug molecules that contain ester or amide linkages in their structure. In the second phase, metabolism primarily involves conjugation reactions in which the drug or its first phase metabolites are coupled with endogenous substances such as glucuronic acid, sulfate, or glycine. These synthetic reactions yield water-soluble metabolites that are more readily excreted via the kidneys in urine and the liver in bile compared with those produced in non-synthetic reactions.

**Figure 2:**
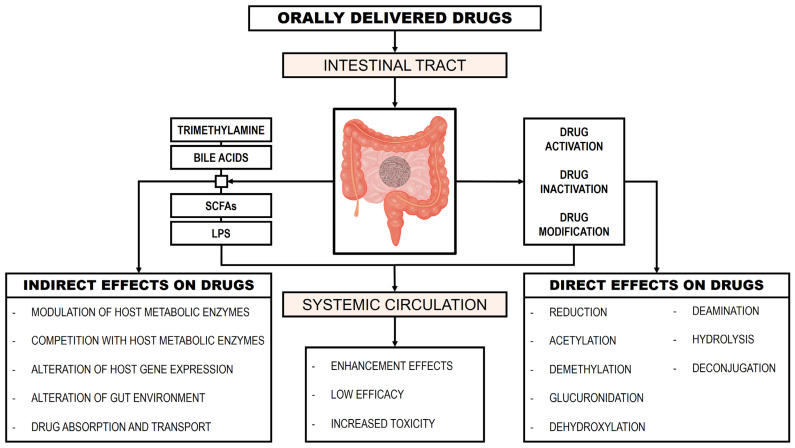
Influence of the gut microbiome on drug metabolism.

However, the majority of orally administered hydrophilic drugs migrate from the upper to the lower intestinal tract, where the GM metabolizes them into hydrophobic metabolites. This transformation facilitates their absorption from the gastrointestinal tract into the bloodstream [22]. Figure 2 presents the influence of the GM on drug metabolism (modified from [22]).

### Drug Biotransformation by the Gut Microbiome

Drug metabolism depends not only on the host, but also on the GM, and this specific field has been termed pharmacomicrobiomics [25]. Biotransformation consists of the conversion of chemical substances within a biological system including microorganisms present in several human microbiomes [174,175]. This microbial biotransformation is produced by microbial exoenzymes that convert low molecular weight organic compounds into analogous compounds by oxidation, reduction, hydrolysis, condensation, isomerization, unsaturation, or by the introduction of heteroatoms [176].

In the case of psychostimulants, the presence of microbial esterases is involved in the biotransformation of cocaine. For example, the *Delftia* (formerly *Comamonas*) *acidovorans* strain MBLF, *Pseudomonas fluorescens* strain MBER, *Rhodococcus* spp. strain MB1, and *Stenotrophomonas* (formerly *Pseudomonas*) *maltophilia* strain MB11L can use cocaine as a carbon and nitrogen source and convert this substance to ecgonine methyl ester and benzoic acid, catalyzed by the enzyme cocaine esterase [177,178,179]. In addition, bacterial carboxylesterases were isolated from cultures of *Bacillus licheniformis* strain ATCC14580 and *B. subtilis* subsp. *subtilis* strain 168 [180]. These enzymes catalyzed the hydrolysis of cocaine to benzoylecgonine and methanol, similar to human liver carboxylesterase hCE1 and hCE2. These enzymes have been used to treat acute cocaine toxicity [181,182,183,184,185]. The microbial biotransformation of amphetamines and related compounds has also been explored in several studies. Amphetamine has been shown to be biotransformed by *Mycobacterium smegmatis* [186]. Additionally, strains of the fungus *Cunninghamella echinulata* were able to metabolize 3,4-methylenedioxy-N-methylarnphetamine (MDMA) and 3,4-methylenedioxyamphetamine (MDA) [187]. Moreover, amphetamine has been observed to bind to tyramine oxidase from the *E. coli* strain present in the human GM [188].

Several opioids can undergo microbial biotransformation through a diverse range of microorganisms. *Pseudomonas putida* strain M10 has been shown to metabolize morphine and codeine via the enzyme morphine dehydrogenase [189], which catalyzes their oxidation into morphinone and codeinone, respectively [190]. These intermediates are further converted by the enzyme morphinone reductase, leading to the formation of hydromorphone and hydrocodone [191,192,193], with hydromorphone also being converted into dihydromorphine [191,193]. The enzyme (3-17)-hydroxysteroid dehydrogenase from *Comamonas* (formerly *Pseudomonas*) *testosteroni* has also been reported to be an effective catalyst in the bacterial biotransformation of morphine to morphinone, a key intermediate in hydromorphone biosynthesis [191]. Another microbial biotransformation described for morphine is the oxidation of this substance by the enzyme laccase, which is present in Ascomycetes, Deuteromycetes, and Basidiomycetes fungi [194] as well as in the endospore of *B. subtilis* [195]. Codeine undergoes biotransformation by *P. putida* strain M10, resulting in the production of codeinone, hydrocodone, dihydrocodeine, and 14β-hydroxycodeine [196]. In addition, *Streptomyces griseus* has been reported to metabolize codeine into norcodeine [197]. *Rhizobium radiobacter* strain R89-1 also facilitates the biotransformation of codeine, yielding hydroxylated metabolites such as 14-hydroxycodeine, 14-hydroxycodeinone, 14-hydroxy-7,8-dihydrocodeine, and 14-hydroxy-7,8-dihydrocodeinone [198]. Moreover, the cyanobacterium *Nostoc muscorum* can metabolize codeine to 6-acetylcodeine, oxycodone, norcodeine, and morphine [199]. The microbial degradation of heroin has been demonstrated in *Rhodococcus* sp. strain H1, which converts the drug to 6-monoacetylmorphine (6-MAM), followed by further metabolism into morphine [200]. Fungi belonging to the genus *Cylindrocarpon didymium* produce biotransformation mediated by the oxidation of morphine to 2,2′-bimorphine [201]. Other fungal species of the genus *Cunninghamella*, such as *C. bainieri*, *C. bertholletiae*, *C. blakesleeana* and *C. echinulata* [202,203], produce the N-demethylation of codeine to norcodeine. In contrast, morphine and oxymorphone showed no biotransformation after incubation with *C. echinulata*, *Curvularia lunata*, *Helicostylum piriforme*, *Sporotrichum sulfurescens*, *Trametes cinnabarina*, and *T. sanguinea* [202].

Akhtar et al. [204] reported that a large number of microbial species can biotransform the 9-tetrahydrocannabinol (Δ^9^-THC) molecule present in cannabinoids by microbial hydroxylation, leading to the formation of a range of mono- and dihydroxylated derivatives [205] such as the fungi *Fusarium nivale*, *Gibberella fujikuroi*, and *Thamnidium elegans* [206]. The biotransformation of Δ^9^-THC, Δ^8^-tetrahydrocannabinol (Δ^8^-THC), cannabidiol, and cannabinol through the partial oxidation of the n-pentyl side chain has been demonstrated in the filamentous fungus *Syncephalastrum racemosum* strain ATCC18192 and the bacterium *Rhodococcus* (formerly *Mycobacterium*) *rhodochrous* strain ATCC 19067 [207,208]. Table 3 presents a list of the major microbial enzymes and the corresponding genes involved in the transformation processes of various drugs.

## 6. Discussion

SUDs significantly affect the physical and mental health of individuals, making it a global public health issue that affected more than 284 million people aged 15–64 years old worldwide in 2020 [218]. At present, the treatment of SUDs mainly depends on psychological withdrawal and drug substitution, but the low efficacy and side effects of these therapies have led to the search for new treatments. The response to addiction pharmacotherapy is complex and depends on genetic, biological, environmental, and social factors [219]. Based on the fact that the dopaminergic system plays a critical role in the pharmacotherapy for addictions, an understanding of the role of the variation in genes involved in this system can be used as a personalized therapy for SUDs, which is one of the main focus of pharmacogenetics. Polymorphisms of several genes have been found to moderate the effects of the pharmacotherapy of alcohol, opioid, and cocaine SUDs (Table 2). However, the review of the development of pharmacogenetic biomarkers for pharmacotherapy personalization shows that the evidence for these biomarkers is still exiguous [220]. In this regard, despite the promising potential of these approaches, only a few treatments have been approved by the FDA and EMA for SUDs including acamprosate, disulfiram, methadone, and naltrexone [221].

Genetic factors alone are not sufficient to explain the individual variability in drug response, and additional factors such as the role of the GM may also be pivotal [74]. Several GWASs have been conducted to investigate the interplay between human genetic variation and the composition of the GM as well as their potential influence on drug addiction [222]. As the field of genome–microbiome interactions is still in its early stages, metagenome-wide association studies (MGWASs) hold promise for further uncovering complex associations, supported by resources such as the Human Microbiome Project and advancements in bioinformatics tools [223]. However, GWASs have certain limitations: (i) microarrays contain a limited number of preselected SNPs [224]; (ii) statistical methods for analyzing multimarker genetic effects in large databases are not easy to develop and implement [225]; (iii) these types of analyses often suffer from small sample sizes combined with the need for significant multiple testing correction [226]; and (iv) the effect of rare and unknown genetic polymorphisms should not be overlooked, as different variants in specific pathways may be responsible for relevant effects in common diseases [227]. Furthermore, the requirement for genome-wide corrections in GWASs requires large sample sizes, often involving thousands of subjects, which may result in the inability to detect variants with small but genuine effects [228].

In 2010, Rizkallah et al. [229] outlined three potential implications of pharmacomicrobiomics for the decade from 2010 to 2019. These included the development of personalized probiotics and phage therapies, both of which are currently being applied to mitigate the adverse effects of chemotherapy and treat persistent infections. However, the proposed resistome scanning or tissue mapping has not yet been adopted as a clinical strategy. Aziz et al. [230] identified three critical challenges in pharmacomicrobiomics that could drive clinical innovations: (i) the systematic implementation of high-throughput microbiome screening; (ii) the application of phage-based precision microbiome engineering and editing; and (iii) the integration of pharmacomicrobiomics into clinical practice. Moreover, these authors predict an increase in pharmacomicrobiomic and pharmacogenomic testing, along with the development of clinical guidelines for key drug classes. We argue that such advancements will mark a critical turning point in the progression of precision medicine.

Pharmacomicrobiomics is a rapidly evolving discipline that aims to understand the complex interactions between the GM, the host, and drugs, with implications for pharmacokinetics, pharmacodynamics, drug metabolism, bioavailability, and potential drug toxicity [26]. While significant advances have been made in this field, several challenges persist, particularly with regard to medical drugs, although they can also apply to drugs of abuse. These challenges include (i) the lack of standardized methodologies in sample collection, sequencing, and data analysis in pharmacomicrobiomics studies, which complicates the comparison of results across different studies and hinders the development of clinical guidelines; (ii) the GM is a complex and dynamic system influenced by intrinsic and extrinsic factors (e.g., diet, lifestyle, host genetics, and age) that may confound the interpretation of results; (iii) inter-individual variability in the effects of drug–GM interactions must be considered; (iv) there is a lack of a comprehensive understanding of the genetic and biochemical mechanisms underlying drug biotransformation; (v) although the potential clinical applications of pharmacomicrobiomics are promising, the development of microbiota-based biomarkers of drug response will require large-scale validation studies and regulatory approval; (vi) many pharmacomicrobiomics studies often have limited sample sizes, which may restrict their statistical power and generalizability; and (vii) there is considerable variability in sequencing methods and molecular tools, making it difficult to compare results across studies [35].

To address these limitations, several strategies are expected to be implemented in the future including the use of: (i) new databases for pharmacomicrobiomics studies [35]; (ii) next-generation meta-omics analysis and high-throughput sequencing techniques such as 16S ribosomal RNA microbial profiling, DNA microarrays, metatranscriptomics, metabolomics, and shotgun metagenomics [231]; (iii) machine learning, a subfield of artificial intelligence that allows machines to acquire knowledge from data without explicit programming [232]; (iv) the development of predictive models and software to simulate drug–microbiota interactions, thereby reducing the research time and costs [233]; and (v) psychobiotics, including probiotics, prebiotics, synbiotics, and postbiotics, which may have the potential to modify the composition and activity of the GM, influencing its interaction with drugs and altering their effects on the host [234].

Finally, in the context of this discussion, it is worth mentioning the emerging field of pharmacotranscriptomics, which explores the relationship between variations in the transcriptome and the pharmacokinetics and pharmacodynamics of drugs. More specifically, it aims to identify interindividual differences, facilitate biomarker discovery, and assess drug efficacy, expanding beyond the scope of traditional pharmacogenomics [235]. In fact, two main strategies are commonly employed and shared by both disciplines: one focuses on analyzing candidate pharmacogenes or pharmacotranscripts, while the other involves comprehensive GWASs or transcriptome-wide association studies (TWASs) to uncover new markers [236]. Although studies based on candidate genes or transcripts provide strong statistical power, they are limited in their ability to discover novel genes or transcripts. On the other hand, GWASs/TWASs are more effective for identifying new relevant markers, but they may present reduced statistical power due to the large number of independent tests. Recent applications of pharmacotranscriptomics include the profiling of resistant triple-negative breast cancer cells treated with lapatinib and berberine as well as analyzing gene networks involved in ferroptosis regulation in cancer [237,238]. Despite pharmacotranscriptomics showing substantial advancements, its direct clinical applications to SUDs are still limited, with the absence of studies on drugs of abuse underscoring this current gap within the field. Furthermore, the integration of gene expression profiling into clinical practice for personalized therapies remains in the developmental phase, lacking the widespread application seen in pharmacogenomics. Nevertheless, as research progresses, pharmacotranscriptomics holds significant potential for contributing to the personalized treatment of SUDs.

## 7. Conclusions

This review highlights the significant role of the GM and its metabolites in various stages of drug addiction including drug seeking, reward, and biotransformation. Identifying pharmacological targets for the treatment of SUDs remains challenging. Therefore, the advancement of precision medicine and the development of biomarkers capable of monitoring drug responses offer valuable opportunities for the development of pharmacotherapies for SUDs. Progress in the pharmacogenomics of SUDs has provided biological insights into the pharmacological needs associated with common genetic variants in drug-metabolizing enzymes.

We conclude that integrating pharmacogenomics with pharmacomicrobiomics will form a crucial foundation for significant advances in precision and personalized medicine, representing a promising field that requires further exploration and enhanced focus in both future research and clinical practice, with the potential to improve patient outcomes, optimize treatment strategies, reduce adverse drug reactions, and lower healthcare costs in the management of SUDs.

## Figures and Tables

**Table 1:** Differences between pharmacogenomics and pharmacomicrobiomics.

Concepts	Pharmacogenomics	Pharmacomicrobiomics
Independent variables	Inherited variations in the human genome or epigenetic modifications.	Changes in microbial composition as well as in the microbial genome, transcriptome, and proteome.
Intraindividual variations	The human genome is a relatively stable entity, with only rare mutations occurring throughout an individual’s lifetime.	The microbiome and its composition vary over time, across different locations, and within an individual due to physiological influences. While subtle, continuous variations arise from intrinsic factors, significant changes can result from dietary shifts and other environmental influences.
Interindividual variations	The study of genetic and genomic variations explains inter-individual differences in drug response. Allelic variations in drug-metabolizing enzymes, such as cytochromes, are among the most extensively investigated. Other allelic variations that may influence drug action include those affecting transporters, target molecules, or receptors.	Initial studies clustered GM profiles into enterotypes. Later research revealed a gradient or continuum of types that can be described using certain β-diversity metrics. Another approach to assessing inter-individual differences focuses on functional rather than microbial profile-based classifications, describing functional clusters or metabotypes.

**Table 2:** Drug therapies used in pharmacogenetic studies and genetic variations.

Pharmacotherapy	Substance	Gene	Variants	Effects
Acamprosate	Alcohol	*DRD2*	rs6277, rs6275, and rs1799978	Reward-related activation [46]
		*GATA4*	rs13273672	Withdrawal and dependence [47]
Baclofen	Alcohol	*GABBR1*	rs29220	Relapse and follow-up consumption [48]
Bromocriptine	Alcohol	*DRD2*	rs6277, rs6275, and rs1799978	Reward-related activation [46]
Naltrexone	Alcohol	*OPRM1*	rs1799971	Reduced consumption and produced lower relapse rates [49,50]
Olanzapine	Alcohol	*DRD4*	Exon 3 VNTR	Craving and related responses [51]
Ondansetrom	Alcohol	*SLC6A4*	5-HTTLPRVNTR	Reduction in craving and heavy drinking [52]
Topiramate	Alcohol	*GRIK1*	rs2832407	Reduced rate of heavy drinking [53]
Disulfiram	Alcohol	*ANKK1*	rs1800497 (*TaqI A*)	Dependence in females [54]
Disulfiram	Cocaine	*DRD2*	rs6277, rs6275, and rs1799978	Reward-related activation [46]
		*ANKK1*	rs1800497	Drug dependence [55]
		*DBH*	rs1611115	Reduction in drug use [56]
		*ADRA1A*	rs1048101	Reduced signaling efficiency [57]
		*TPH2*	rs4290270	Increased serotonin levels [58]
Doxazosin	Cocaine	*DBH*	rs1611115	Reduction in drug use [59]
Cocaine vaccine	Cocaine	*OPRK1*	rs6473797	Reduced the rate of dopamine [60]
		*DBH*	rs1611115	Reduction in drug use [61]
Methadone	Opioids	*DRD2*	rs6277, rs6275, and rs1799978	Maintenance therapy for opioid addiction [62]
		*ABCB1*	rs1045642, rs1128503, and rs2032582	Multidrug resistance [63]
		*BDNF*	rs988748, rs1967554, rs2030324, rs2239622, rs7127507, rs11030118, and rs11030119	Maintenance therapy for opioid addiction [64]
		*ARRB2*	rs2036657, rs3786047, and rs1045280	Maintenance therapy for opioid addiction [65]
		*CYP2B6*	rs2279343 and rs3745274	Maintenance therapy for opioid addiction [66]
		*CYP2D6*	rs1065852	Maintenance therapy for opioid addiction [42,67]
		*MYOCD*	rs1714984	Maintenance therapy for opioid addiction [68]
		*GRM6*	rs953741	Maintenance therapy for opioid addiction [68]
		*NECTIN4*	rs3820097, rs4656978, and rs11265549	Maintenance therapy for opioid addiction [69]
		*KCNJ6*	rs2070995	Opioid receptor signaling and reward processing [70]
		*OPRM1*	rs558025 and rs2075572	Maintenance therapy for opioid addiction [71]
		*GABRB2*	rs3219151	Maintenance therapy for opioid addiction [72]
Buprenorphine	Opioids	*OPRD1*	rs529520 and rs581111	Maintenance therapy for opioid addiction [73]

*ABCB1*: ATP-binding cassette, subfamily B (MDR/TAP), member 1 gene; *ADRA1A*: α1A-adrenoceptor gene; *ANKK1*: ankyrin repeat and kinase domain-containing 1 protein gene; *ARRB2*: β-arrestin 2 gene; *BDNF*: brain-derived neurotrophic factor gene; *CYP2B6*: cytochrome P450, family 2, subfamily B, polypeptide 6 gene; *CYP2D6*: cytochrome P450, family 2, subfamily D, polypeptide 6 gene; *DBH*: dopamine β-hydroxylase gene; *DRD2*: dopamine receptor D2 gene; *DRD4*: dopamine receptor D4 gene; *GABBR1*: γ-aminobutyric acid type B receptor subunit 1 gene; *GABBR1*: γ-aminobutyric acid type B receptor subunit 2 gene; *GATA4*: GATA binding protein 4 gene; *GRM6*: glutamate receptor metabotropic 6 gene; *GRIK1*: glutamate ionotropic receptor kainate-type subunit 1 gene; *KCNJ6*: potassium inwardly rectifying channel, subfamily J, member 6 gene; *MYOCD*: myocardin gene; *NECTIN4*: nectin cell adhesion molecule 4 gene; *OPRD1*: δ-opioid receptor gene; *OPRK1*: κ-opioid receptor gene; *OPRM1*: μ-opioid receptor gene; *SLC6A4*: serotonin transporter gene; *TPH2*: tryptophan hydroxylase 2 gene.

**Table 3:** Microbial enzymes and genes involved in the biotransformation of drugs of abuse.

Substance	Enzyme	Gene	Microorganism	Microbial Product	Ref.
Cocaine	Cocaine esterase	*cocE*	*Rhodococcus* spp.	Ecgonine methyl ester and benzoic acid	[177]
	Carboxyesterase	*CES1*, *CES2*	*Bacillus* spp.	Benzoylecgonime and methanol	[180,209]
Amphetamine	Tyramine oxidase	*tynA*, *tynB*	*Escherichia coli*	R-(-) enantiomers	[188]
Morphine	Morphine dehydrogenase	*morA*	*Pseudomonas putida*	Dehydromorphine	[210]
	Morphinone reductase	*morB*	*P. putida*	Hydromorphine	[211]
	17-Hydroxysteroid dehydrogenase 3	*HSD17B3*	*Comamonas testosteroni*	Morphimone	[212]
	Laccase	*lcc1*, *lcc2, and lcc3*	*Coprinus cinereus*	Morphine-glucuronide derivatives	[213]
	Laccase	*cotA*	*Bacillus subtilis*	Di-, oligo-, and polymers of drug	[214,215]
Codeine	Morphine dehydrogenase	*morA*	*P. putida*	Codeinone	[190,210]
	Cytochrome P450 monooxygenases	*CYP2D6*	*Streptomyces* spp.	Norcodeine	[216]
Heroine	Heroine esterase	*her*	*Rhodococcus* spp.	6-monoacetylmorphine	[217]
